# Reactivity of human AGO2 monoclonal antibody 11A9 with the SWI/SNF complex: A case study for rigorously defining antibody selectivity

**DOI:** 10.1038/s41598-017-07539-4

**Published:** 2017-08-04

**Authors:** Roderick A. P. M. van Eijl, Teun van den Brand, Luan N. Nguyen, Klaas W. Mulder

**Affiliations:** 1 Department of Molecular Developmental Biology, Radboud University Nijmegen, Nijmegen, The Netherlands; 2 Molecular Biology, Radboud Institute for Molecular Life Sciences, Nijmegen, The Netherlands

## Abstract

In this study, we originally aimed to characterize the potential role of Argonaute 2 (AGO2) in the nucleus, a key protein of the miRNA machinery. We combined Chromatin Immunoprecipitation (ChIP) with high throughput sequencing (ChIP-seq) and quantitative mass spectrometry (ChIP-MS) using the broadly used AGO2 11A9 antibody to determine interactions with chromatin and nuclear proteins. We found a previously described interaction between AGO2 and SWI/SNF on chromatin with ChIP-MS and observed enrichment at enhancers and transcription start sites using ChIP-seq. However, antibody specificity issues can produce misleading results for ChIP, RNA-seq and Mass spectrometry. Therefore, we developed a CRISPR/Cas9 engineered AGO2^−/−^ HEK293T cell line to validate our findings. ChIP-qPCR and immunoprecipitation combined with MS (IP-MS) showed that the 11A9 antibody associates with chromatin and SWI/SNF in the absence of AGO2. Furthermore, stoichiometry, IP-MS and co-IP analysis suggests a direct interaction of this antibody with SMARCC1, a component of the SWI/SNF complex. For this reason, particular care should be taken in performing and interpreting experiments in which the 11A9 antibody is used to study a nuclear role of AGO2.

## Introduction

MicroRNAs (miRNAs) are known for their role in fine tuning the expression of genes through modulation of mRNA levels and protein translation^1^. Classical miRNA action takes place in the cytoplasm involving sequence specific targeting of mRNAs leading to degradation and/or inhibition of translation^2^. AGO2 is a key protein of the cytoplasmic RNAi machinery and directly binds the miRNAs.

Apart from a cytoplasmic function, RNAi machinery components are also found in the nucleus of mammalian cell^3–5^. It is shown that AGO2 can shuttle between the nucleus and the cytoplasm in association with small RNAs and most proteins of the cytoplasmic RNAi machinery^6–9^. In addition, AGO2 has been reported to stabilize small RNAs in the nucleus and most of these small RNAs were end-processed by DICER, which supports the idea that miRNAs are transported back into the nucleus by the RNAi machinery^10^. In other species than mammalian, there is more evidence for a nuclear RNAi mechanism^11–13^ and in Drosophila, AGO2 association with specific genomic binding sites has been reported as well^14^. Recently, the potential role of AGO2 in the nucleus has gained more attention and different nuclear mechanisms in mammalian cells involving AGO1 and AGO2 have been described and reviewed^15^. These mechanisms include transcriptional gene silencing^16–19^, gene activation^20–23^ and alternative splicing^24, 25^. In particular, one report described an interaction of AGO2 with the chromatin remodelling complex SWI/SNF^26^. This interaction was identified using mass spectrometry (MS) analysis of nuclear proteins immuno-precipitated with the AGO2 11A9 antibody and co-immuno-precipitations (co-IPs) were done for confirmation. However, this interaction is somewhat controversial, as another report showed conflicting results with a different AGO2 antibody (Abcam 57113) and with a FLAG-tagged AGO2^9^. Here, they also performed immunoprecipitations (IP) combined with MS and Western blot on nuclear and cytoplasmic extracts. Using this approach they identified proteins interacting with AGO2 in the nucleus as well as in the cytoplasm, but no interaction with SWI/SNF. In addition, the report that originally described the AGO2 11A9 antibody already noted that this antibody recognizes a nuclear protein with unknown identity^3^.

Regardless of the controversial interaction of AGO2 with SWI/SNF, it seems probable that AGO2 associates with chromatin, yet it remains unclear how and where on the genome it is bound. Our study originally aimed to further investigate this by combining Chromatin Immunoprecipitation (ChIP) with quantitative MS and high-throughput sequencing^27^ in primary human epidermal stem cells and the HEK293T cell line. Using this approach, we chose the rat monoclonal 11A9 antibody against human AGO2 which is commonly used and shown to be non-reactive with other Argonaute proteins^3^. Applications for which this antibody has been used, include immunofluorescence^3^, co-immunoprecipitation (IP)^3^, nuclear IP combined with quantitative Mass spectrometry (MS)^26^, Chromatin Immunoprecipitation (ChIP) with qPCR^25^ and RNA-IP for AGO2 associated RNAs (AGO2-RIP)^25, 28^. The suspicious interactions with unknown nuclear proteins together with the diverse uses of this antibody, and especially the use in our strategy requires good characterization^3, 29, 30^. Although this may seem obvious, no in-depth characterisation of this antibody has been described, especially for non-standard applications such as ChIP-seq and ChIP/IP-MS. We used the AGO2 11A9 antibody in ChIP-MS and identified the previously reported interaction between AGO2 and members of the SWI/SNF complex. Furthermore, we performed ChIP-seq analysis in parallel that revealed genomic sites presumably enriched for AGO2 occupancy. To rigorously validate these observations, we engineered a CRISPR/Cas9 mediated AGO2^−/−^ HEK293T line. We found that the 11A9 antibody strongly enriched for SWI/SNF factors with IP-MS, even in the absence of AGO2 protein. Furthermore, ChIP-qPCR analysis of loci enriched in our ChIP-seq dataset indicated that this signal was independent of AGO2. Moreover, stoichiometry analysis in combination with IP-MS of the SWI/SNF complex, as well as reciprocal co-IPs from WT and AGO2^−/−^ lysates, identify a likely cross-reactivity of the 11A9 antibody with the SWI/SNF component SMARCC1.

## Results

### ChIP-MS using anti-AGO2 11A9 suggests interactions between AGO2 and SWI/SNF factors

**Figure 1:**
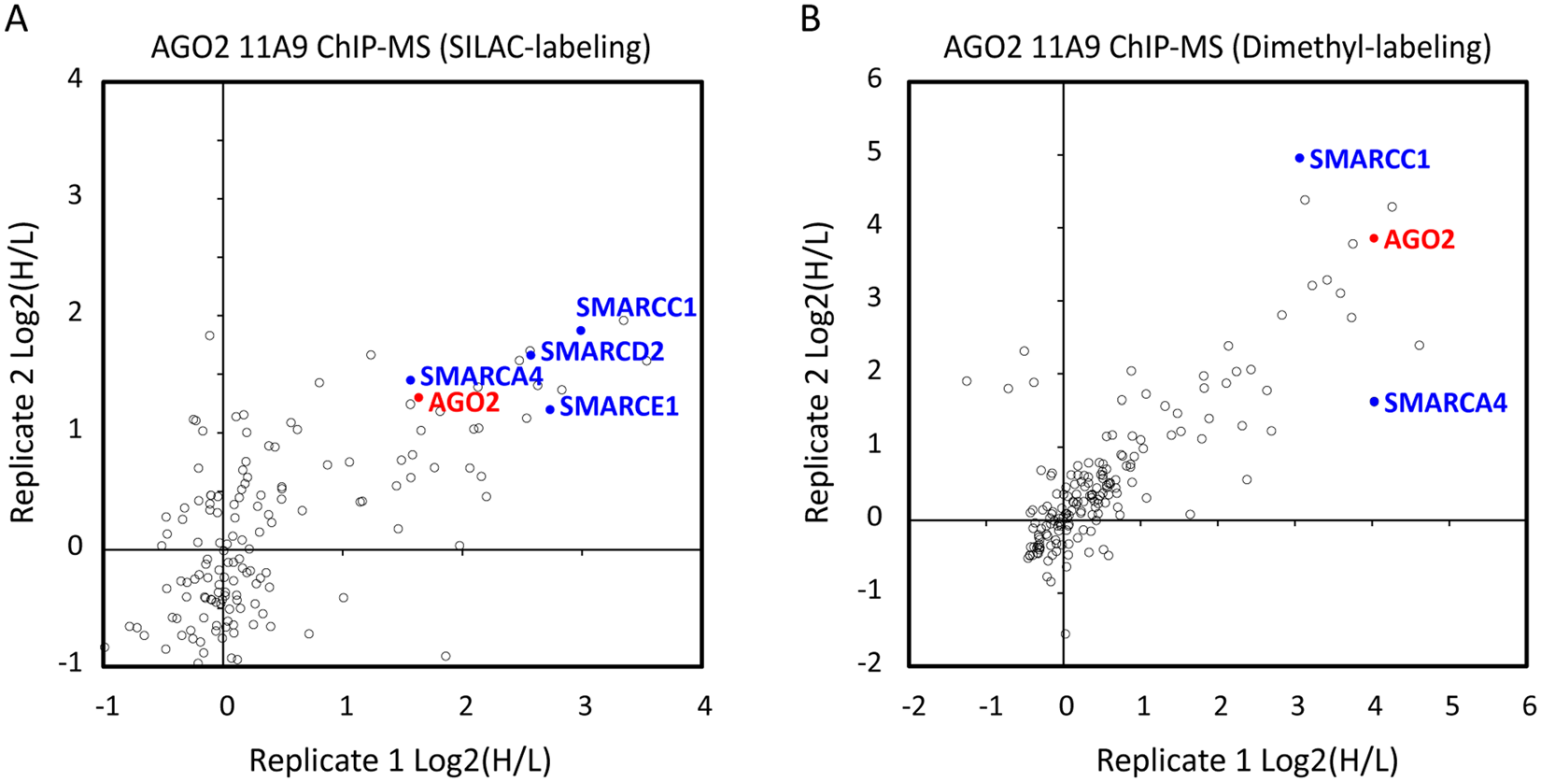
Anti-AGO2 ChIP-MS in epidermal stem cells suggests AGO2 interaction with SWI/SNF complex proteins. (**A**) ChIP-MS with SILAC labeling of AGO2 11A9 antibody and anti-FLAG control. (**B**) ChIP-MS with dimethyl-labeling of AGO2 11A9 antibody and IgG isotype control. In red anti-AGO2 11A9 antigen AGO2 and in blue SWI/SNF complex proteins.

To investigate a potential nuclear role of AGO2 in epidermal stem cells, we aimed to identify novel physical interactions within this cellular compartment. To allow identification of potential AGO2 interactors on chromatin and to stabilize transient protein-protein interactions, we combined formaldehyde cross-linking and chromatin immunoprecipitation (ChIP) using the anti-AGO2 11A9 antibody with quantitative mass spectrometry (MS)^27^. Using stable isotope labelling by amino acids in cell culture (SILAC), we found that components of the SWI/SNF complex, including SMARCA4, SMARCC1, SMARCD1 and SMARCE1 co-purified with AGO2 (Fig. 1A). These results were confirmed with a second quantitative MS technique using dimethyl-labelling^31^ (Fig. 1B). These findings are in line with previous observations^26^ and suggest that AGO2 may interact with chromatin and the SWI/SNF complex.

**Figure 2:**
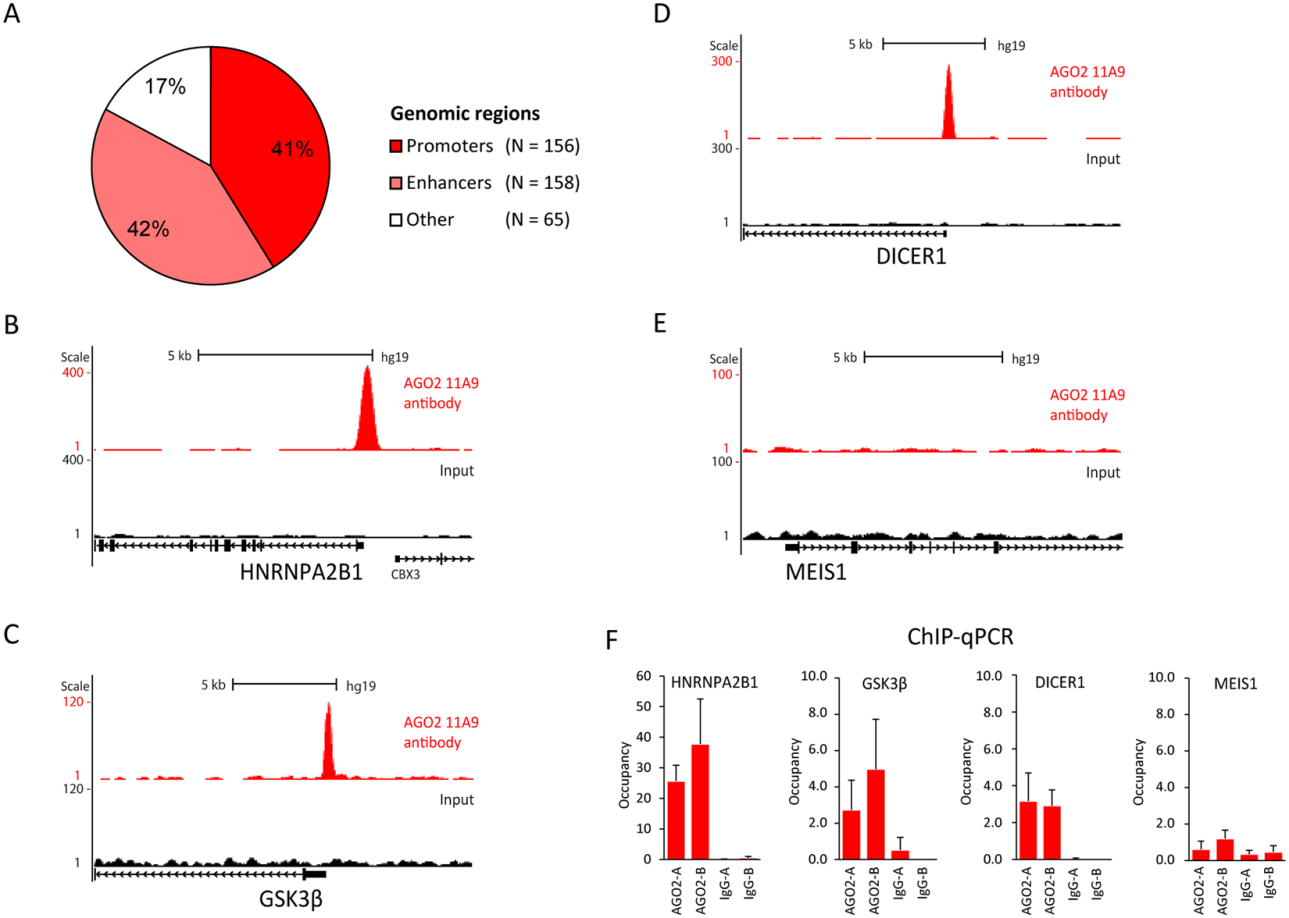
Chromatin immunoprecipitation with anti-AGO2 11A9 enriches promoters and enhancers in epidermal stem cells. (**A**) Alignment with promoters/enhancers regions with 379 best peaks obtained with MACS peak calling. (**B**–**E**) ChIP-seq in epidermal stem cells at the transcription start site of the indicated loci. (**F**) ChIP-qPCR in epidermal stem cells at the indicated loci. Meis1 is a negative region and occupancy represents enrichment over a heterochromatic region. A and B represent biological replicates and error bars represent standard deviations of 3 technical replicates.

In parallel with the quantitative MS analysis in epidermal stem cells, AGO2 association with chromatin was investigated with Chromatin Immunoprecipitation followed by high-throughput sequencing (ChIP-seq). We identified 379 high confidence peaks (MACS p < 10^−8^), most of which were located in close proximity to known genes. To investigate their genomic distribution, we aligned the peaks with a chromatin state track generated in normal human epidermal keratinocytes (NHEK) from the ENCODE project^32^. This revealed that 42% of the peaks overlap with active enhancer regions and 41% with active promoters, suggesting a potential role for AGO2 in transcriptional gene regulation (Fig. 2A). The *HNRNPA2B1*, *DICER1* and *GSK3β* loci are examples of peaks at transcription start sites (TSSs) representing a range of occupancy levels (Fig. 2B–D, note the differences in scales on Y-axes). We confirmed enrichment at these sites in independent duplicate ChIP-qPCR experiments (Fig. 2F). Moreover, *Meis1* is an active locus based on H3K4me3 enrichment (500-fold over background, data not shown), but negative for AGO2 (Fig. 2E and F). This shows that the AGO2 11A9 antibody does not simply enrich for active, more accessible loci. Furthermore, ChIP with non-specific IgGs did not show enrichment at any of the loci, indicating that the enrichments are indeed specific for the AGO2 11A9 antibody.

**Figure 3:**
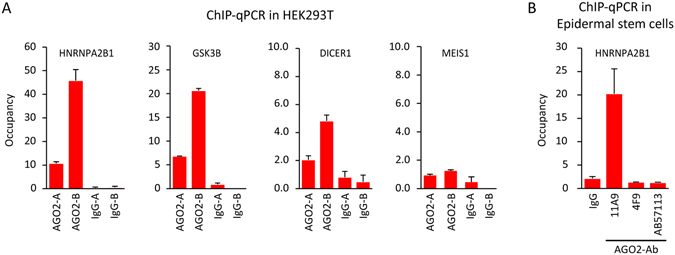
The anti-AGO2 11A9 ChIP enriched loci seem not cell type specific or AGO2 specific. (**A**) ChIP-qPCR in HEK293T cells at the same loci as in Fig. 1F. Meis1 is a negative region. (**B**) ChIP-qPCR in epidermal stem cells with anti-IgG and 3 different AGO2 antibodies at the HNRNPA2B1 locus. Occupancy represents enrichment over a heterochromatic region, A and B represent biological replicates and error bars represent standard deviations of 3 technical replicates.

Furthermore, the same loci were enriched in an 11A9 ChIP in HEK293T cells, showing that the AGO2 11A9 ChIP enrichments were not cell-type specific (Fig. 3A). Next, we tested two other antibodies against AGO2 (4F9 and ab57113) in ChIP-qPCR (Fig. 3B). This showed that the enrichment at the HNRNPA2B1 locus was exclusively found using the 11A9 antibody. This could mean that this antibody is the only one of the three suitable for ChIP enrichment of AGO2. Alternatively, it could be that the enrichment we observed is the result of cross-reactivity with a chromatin-bound factor, independent of AGO2. This prompted us to generate an AGO2 genetic knock-out cell line, to discriminate between these two possibilities and to rigorously test our findings.

### Generation of a CRISPR/Cas9 engineered AGO2^−/−^ clone to validate anti-AGO2 11A9

**Figure 4:**
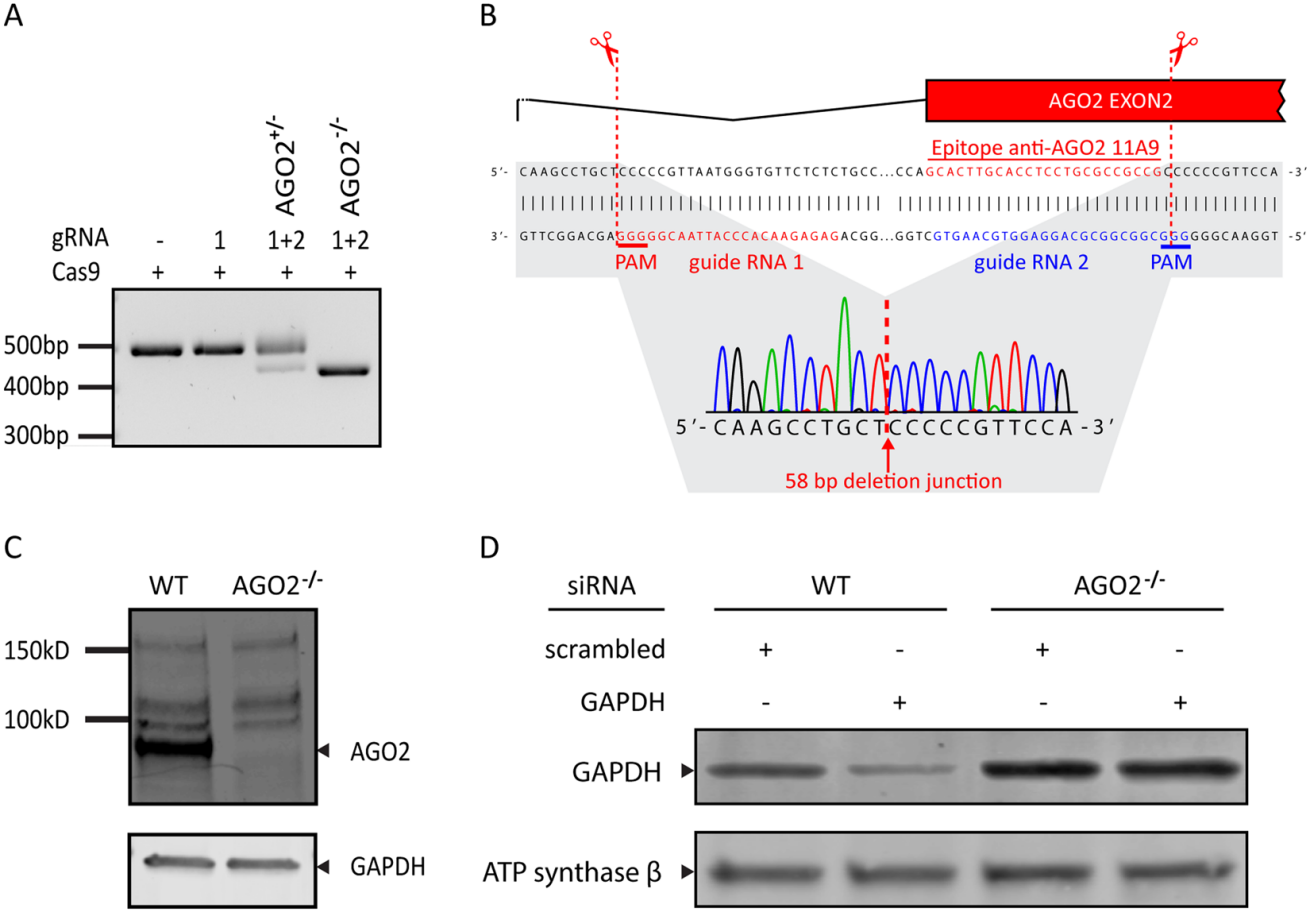
AGO2 11A9 antibody validation with CRISPR/Cas9 introduced AGO2 knock-out. (**A**) Agarose gel showing the genotyping PCR products amplified from the CRISPR/Cas9 targeted region. (**B**) Overview figure displaying the deletion by CRISPR/Cas9 determined with Sanger sequencing. The AGO2 exon2 bar corresponds with the underlying sequence and in red the sequence encoding the epitope of anti-AGO2 11A9. (**C**) AGO2 11A9 stained Western blot loaded with whole cell lysates from wild type and AGO2 knock-out HEK293T cells. GAPDH was stained as loading control. (**D**) Western blot of siRNA treated wild-type and AGO2 knock-out HEK293T extracts. ATP synthase-β was used as a loading control. Uncropped images are shown in the supplementary.

In the literature, conflicting results have been reported for the interaction between AGO2 and SWI/SNF using quantitative IP-MS and co-IPs. One report identified the interaction, as did we, using the 11A9 antibody, although another report did not observe this interaction in co-IP and IP-MS experiments using FLAG-tagged AGO2 or using the AGO2 Abcam 57113 antibody^9, 26^. To validate the use of the AGO2 11A9 antibody in our experiments, we engineered a CRISPR/Cas9 mediated AGO2 knock-out in HEK293T cells. The start of exon 2 AGO2 encodes 8 of the 15 amino acids (MYSGAGPALAPPAPP) of the AGO2 11A9 antibody epitope^3^. To disrupt this epitope sequence, we designed and cloned two CRISPR/Cas9 constructs to delete the beginning of exon 2. Single-cell derived HEK293T clones transfected with these constructs were screened for deletions using a genotyping PCR (Fig. 4A). Two rounds of transfections were necessary to obtain a clone with a deletion in both the AGO2 alleles (AGO2^−/−^). These deletions were further characterized with Sanger sequencing (Fig. 4B), which showed a 58 base pairs (bp) frameshift mutation that were identical in both alleles. Most likely, this occurred through homologous directed repair (HDR) induced by CRISPR/Cas9 in the second round of targeting where the first mutated allele was used as a repair template. Western blot analysis confirmed that the AGO2 protein was no longer expressed in the AGO2^−/−^ clone (Fig. 4C). AGO2 plays an important role in RNA interference (RNAi) using small interfering RNAs (siRNAs) by degrading the target mRNAs via its unique slicer activity. When AGO2 is absent, the introduction of siRNAs into the cell should not lead to degradation of target mRNA and subsequent knock-down of the corresponding protein. To determine whether the AGO2^−/−^ clone constitutes a full functional knock-out, we transfected the AGO2^−/−^ clone and wild-type (WT) HEK293T cells with control or GAPDH directed siRNAs. Upon transfection with GAPDH siRNAs, Western blot analysis showed a decrease of GAPDH protein in WT cells and not in AGO2^−/−^ cells. This indicates that the AGO2^−/−^ clone is indeed a functional AGO2 knock-out (Fig. 4D). Taken together, we created an AGO2^−/−^ clone that can be used to verify our findings with the AGO2 11A9 antibody.

### The 11A9 antibody enriches SWI/SNF factors and chromatin in the absence of AGO2

**Figure 5:**
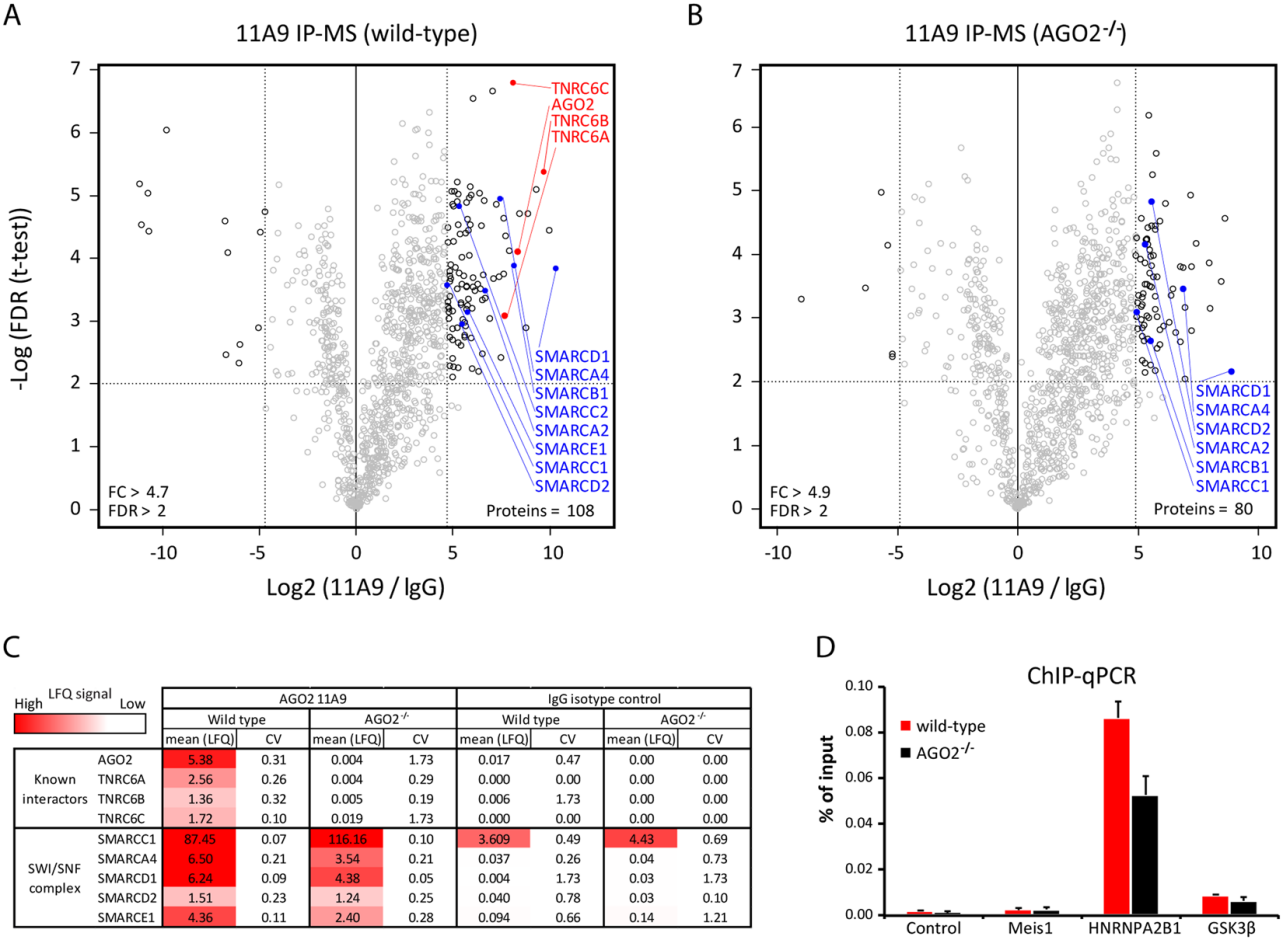
The 11A9 antibody enriches SWI/SNF factors in the absence of AGO2. (**A**,**B**) Label-free quantification of 11A9 antibody associated proteins compared to IgG isotype control. IP-MS experiments were performed in triplicate in wild type and AGO2 knock-out HEK293T cells. For the analysis a two-sample t-test was applied. (**C**) Table with mean Maxquant derived label free quantification (LFQ) intensities (x10) of 3 IP-MS experiments for 11A9 and IgG with HEK293T wild-type and AGO2 knock-out lysates. AGO2 TRNC6A, B and C signals in AGO2 knock-out extracts are the result form imputation. CV represents coefficient of variation. (**D**) ChIP-qPCR in wild type and knock-out HEK293T cells. The control region (heterochromatic site) and Meis1 locus represent negative sites.

To verify the interaction of AGO2 with SWI/SNF factors we performed label-free quantitative (LFQ) IP-MS on WT and AGO2^−/−^ HEK293T lysates using 11A9 and IgG antibodies. These experiments were performed in triplicate to allow outlier identification via statistical analysis. In accordance with the ChIP-MS experiments in epidermal stem cells, we enriched AGO2 and eight SWI/SNF factors from WT lysates using the 11A9 antibody. We also enriched three known interactors of AGO2: TNRC6A, B and C (Fig. 5A). As expected, AGO2, TNRC6A, B and C were not identified with IP-MS on AGO2^−/−^ lysates, confirming the specificity of these common interactors and the known affinity of the 11A9 antibody for AGO2 (Fig. 5B). Surprisingly, SWI/SNF factors were still enriched in the IP-MS on AGO2^−/−^ lysates (Fig. 5B), suggesting cross-reactivity of the 11A9 antibody with one or more of the SWI/SNF factors. The raw LFQ signals of AGO2^−/−^ lysates compared to WT lysates show a strong decrease of signal for AGO2 and its common interactors and no decrease for the SWI/SNF factors (Fig. 5C and Supplementary Dataset 1). Please note that the low LFQ signal for AGO2, TNRC6A, B and C in AGO2^−/−^ lysates are a result of an imputation step included in the data analysis procedure. To verify whether the 11A9 antibody specifically enriches AGO2 on chromatin, we performed ChIP-qPCR using WT and AGO2^−/−^ HEK293T cells to assess the association with the *HNRNPA2B1* and *GSK3β* loci, representing different occupancy levels in our original ChIP-seq dataset. Enrichment at these sites was observed in WT as well as AGO2^−/−^ cells compared to the negative control, indicating that the 11A9 antibody binds chromatin at these loci in the absence of AGO2 (Fig. 5D). We have also performed ChIP-seq with the AGO2 Abcam 57113 antibody in the WT and AGO2^−/−^ cells. However, we did not identify any enriched regions using this antibody (data not shown). Altogether, these results suggest that the 11A9 antibody specifically binds other proteins than AGO2 on chromatin.

### The AGO2 11A9 antibody cross-reacts with SWI/SNF component SMARCC1/BAF155

**Figure 6:**
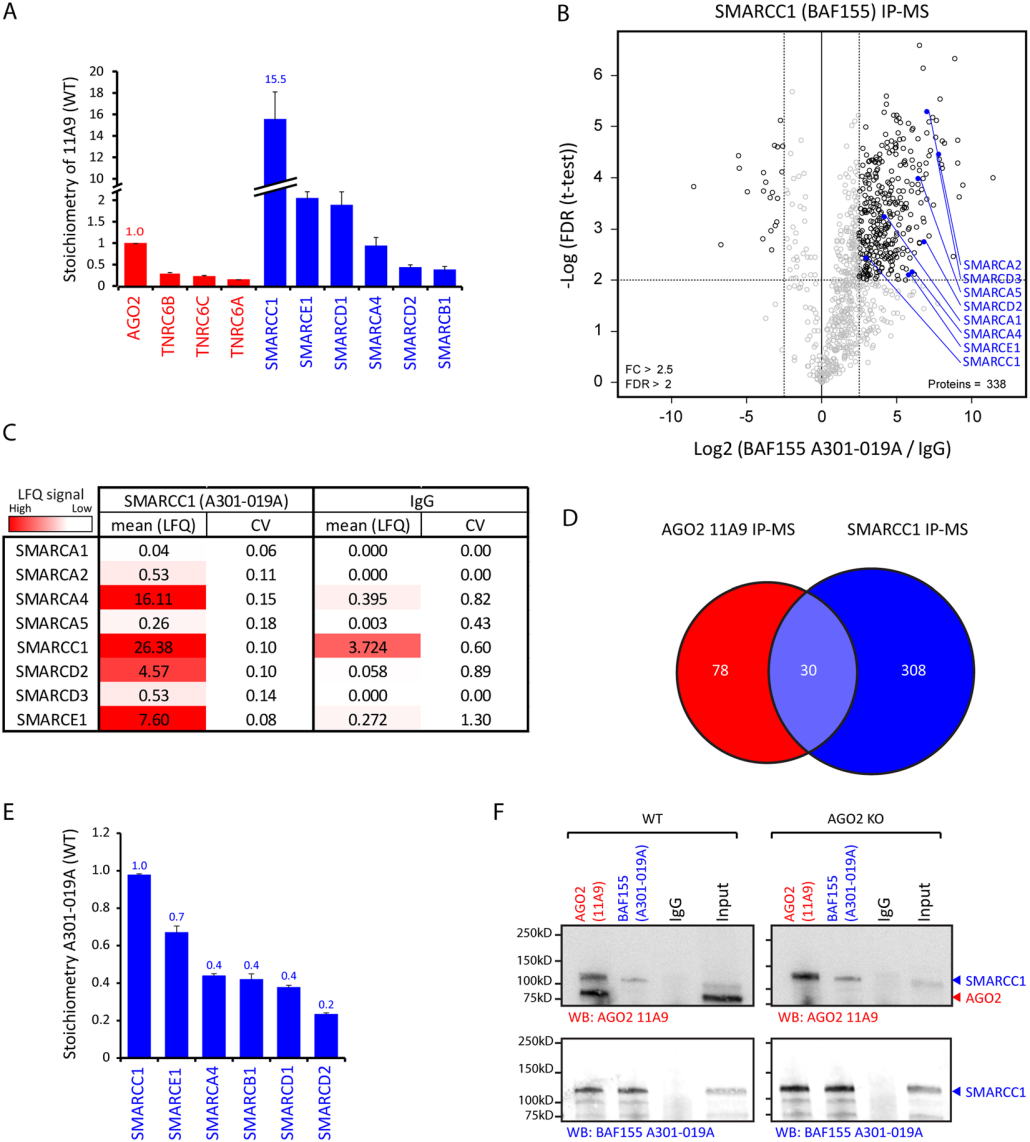
The 11A9 antibody may cross-react with SWI/SNF component SMARCC1/BAF155. (**A**) Stoichiometry analysis in HEK293T with iBAQ values generated with Maxquant normalized with the IgG isotype control. AGO2 is the bait and set at 1 as we used anti-AGO2 (11A9) for the IP. Error bars represent standard deviations of 3 individual IPs. (**B**) Label-free quantification of BAF155 A301-019A associated proteins compared to IgG control. Experiment was performed in triplicate in HEK293T cells. For the analysis a two sample T-test was applied. (**C**) Table with average Maxquant derived label free quantification intensities of 3 IP-MS experiments for BAF155 A301-019A and IgG with HEK293T wild type lysates. Mean LFQ values are times 10 billion and signals from AGO2 knock-out come from imputation. CV represents coefficient of variation. (**D**) Venn diagram representing the number of quantified and significant proteins in the SMARCC1 IP-MS and the AGO2 11A9 IP-MS and proteins that were quantified and significant in both experiments. (**E**) Stoichiometry analysis as in A with SMARCC1 as bait. SMARCC1 was set at 1 as we used anti-BAF155 (A301-019A) for the IP. Error bars represent standard deviations of 3 individual IPs. (**F**) Reciprocal co-IPs with AGO2 11A9 and BAF155 antibodies with anti-IgG as negative control in Wildtype and AGO2 knock-out HEK293T cells. All samples were loaded on the same gel and transferred to the same blot on which both the staining’s were performed with different detection methods. Uncropped images are shown in the supplementary.

After observing that the 11A9 antibody associated with SWI/SNF factors in the absence of AGO2, we mined the IP-MS data to further investigate this cross-reactivity. We applied a previously described algorithm^33^ to determine the stoichiometry of the interactors of the 11A9 antibody, including the bait AGO2 and the SWI/SNF factors that we quantified in our IP-MS experiment. This allowed us to estimate the relative number of interactor protein molecules associated with the 11A9 antibody for each AGO2 protein molecule (Fig. 6A). The known AGO2 interactors TRNC6A, B and C associated with a stoichiometry of 0.1–0.2 per AGO2 molecule, in line with their presumed mutually exclusive interaction with AGO2. In contrast, 15 to 16 copies of the SWI/SNF component SMARCC1 were identified for each AGO2 protein, suggesting that SMARCC1 might be interacting with the 11A9 antibody directly. This high recovery of SMARCC1 with the 11A9 antibody compared to AGO2 itself could potentially also be partially explained by a high abundance of SMARCC1 protein in the lysate. For this reason we looked into the PaxDB protein abundance database for the abundance of AGO2 and SMARCC1 in HEK293T cells^34, 35^. Both proteins had a similarly high abundance based on their number of proteins per million (145 and 169, respectively). According to our stoichiometry analysis and the minor difference in total abundance of both proteins, SMARCC1 seems to bind the AGO2 11A9 antibody more efficiently than AGO2 itself, either directly or indirectly. This is in line with the proposed cross-reaction interaction between SWI/SNF factors and the 11A9 antibody, rather than a *bona fide* protein-protein interaction.

To get an overview of the proteins that are immuno-precipitated by the SMARCC1 antibody, we performed an IP-MS experiment in HEK293T cells. Multiple SWI/SNF factors and other proteins were enriched as interactors (Fig. 6B). Notably, AGO2 was not enriched in these samples. Also note that the number of interacting proteins is rather high due to the use of low stringency buffer conditions in the SMARCC1 IP-MS experiment. Nonetheless, the highest LFQ value was measured for SMARCC1 (Fig. 6C). Ultimately, to see how the SMARCC1 IP-MS relates to the AGO2 11A9 IP, we compared the quantified interactors and the stoichiometry of SMARCC1. These experiments had 30 quantified interactors in common, suggesting that these proteins are identified through an interaction with SMARCC1, rather than with AGO2 (Fig. 6D). In the SMARCC1 IP, the SWI/SNF factors associated with a stoichiometry of 0.2–0.7 per SMARCC1 protein (Fig. 6E), indicating that we purified an intact SWI/SNF complex and that the majority of cellular SMARCC1 resides in this complex and not as a free monomer. In contrast, SMARCC1 was substantially more over-represented in the 11A9 IP-MS experiments (15–16 fold, Fig. 6A). This may be due to the more stringent buffer conditions in the 11A9 IP resulting in lower amounts of co-purified proteins that bind the antibody through protein-protein interactions. This explanation also supports the possibility that SMARCC1 directly interacts with the 11A9 antibody. Finally, we performed reciprocal co-IPs for AGO2 and SMARCC1 in WT and AGO2^−/−^ HEK293T cells (Fig. 6F). In accordance with our previous findings (Figs 5B,C and 6A), SMARCC1 was detected in 11A9 WT and AGO2 KO immuno-precipitates. This provides further proof that the enrichment of SMARCC1 by the 11A9 antibody does not require the presence of AGO2. In line with this, we could not find AGO2 in the reciprocal IPs with a SMARCC1 antibody (Fig. 6F).

Together, the results presented here show that SMARCC1 cross-reacts directly, or indirectly, with the 11A9 antibody, which may lead to erroneous conclusions when using this antibody for nuclear IP-MS or ChIP experiments to investigate AGO2.

## Discussion

In this study, we aimed to address the nuclear role of AGO2 in epidermal stem cells through ChIP-sequencing and MS using the 11A9 antibody. This antibody showed enrichment at enhancer/promoter regions with ChIP-seq and enriched AGO2, SMARCC1 and other SWI/SNF factors with ChIP-MS. The observed interaction between AGO2 and SMARCC1 is in accordance with a previous report in which the interaction was shown using the same antibody^26^. We confirmed these results in HEK293T cells as well. However, by using a CRISPR/Cas9 engineered AGO2^−/−^ HEK293T line, we demonstrated that this interaction does not depend on the presence of AGO2. Indeed, we still observed a strong enrichment for SWI/SNF factors using IP-MS in AGO2 knock-out cells. In addition, ChIP-qPCR enrichment at two representative promoter regions was essentially unaffected after knocking-out AGO2. Previously, the AGO2 interactors have also been addressed with a FLAG-tagged AGO2 and the AGO2 Abcam 57113 antibody. With this antibody and via the FLAG-tagged AGO2, no interaction was observed between AGO2 and SMARCC1 or other SWI/SNF components supporting the idea that SMARCC1 represents an unintended binder of the AGO2 11A9 antibody^9^.

Our stoichiometry analysis for 11A9 antibody interacting proteins showed that SWI/SNF factor SMARCC1 associated 15.5 times more frequent than AGO2. This suggests that SMARCC1 is a likely candidate as direct interactor of the 11A9 antibody. In line with this, we noted that the peptide epitope used to generate the 11A9 antibody is proline rich and that the C-terminus of SMARCC1 contains a very similar, and proline-rich, sequence (QPPPPPPADGVPPPPAPGP). In addition, Rüdel *et al*. (2008), who originally reported the 11A9 antibody, showed an unidentified band on an 11A9 probed Western blot loaded with nuclear extract^3^. Although cross-reactivity on a Western blot is not necessarily representative of cross-reactivity in an immunoprecipitation approach, our data strongly argue that this reported band represents SMARCC1. To determine if SMARCC1 indeed directly binds the 11A9 antibody allowing re-interpretation of our ChIP data, we applied CRISPR/Cas9 on the SMARCC1 locus as well. However, we were not able to engineer a SMARCC1 knock-out line despite repeated attempts and only obtained cell lines with in-frame deletions (data not shown). This might suggest that SMARCC1 knock-out has a negative effect on cell survival or proliferation in HEK293T cells. Therefore, we cannot formally exclude that the SWI/SNF and ChIP-qPCR enrichments we observed in our experiments can be explained by other proteins than SMARCC1 associating with the AGO2 11A9 antibody.

Taken together, our data indicate that the AGO2 11A9 antibody associates with SMARCC1 in IP-MS aproaches using whole lysates or nuclear extracts. This may warrant a careful reevaluation of the published literature making use of this particular antibody. Antibodies in general should be validated properly as they all have their own artefacts. Here, we strongly recommend verifying nuclear AGO2 interactions identified with the 11A9 antibody using an AGO2 knock-out line, tagged-AGO2 proteins or alternative antibodies.

## Materials and Methods

### Cell culture, transfection, clonal selection and knock-down

Primary pooled human epidermal stem cells derived from foreskin were obtained from Lonza. Cells were cultured and expanded as previously reported^36^. Briefly, cells were seeded on a feeder layer of J2-3T3 cells in FAD medium (Ham’s F12 medium/Dulbecco’s modified Eagle medium (DMEM) (1:3) supplemented with 10% batch tested fetal calf serum (FCS) and a cocktail of 0.5 µg/ml of hydrocortisone, 5 µg/ml of insulin, 0.1 nM cholera enterotoxin, and 10 ng/ml of epidermal growth factor. J2-3T3 cells were cultured in DMEM containing 10% bovine serum. HEK293T cells were cultured in DMEM supplied with 10% fetal bovine serum. All media were supplemented with 1% penicillin/streptomycin antibiotics.

For transfections HEK293T cells were incubated 4 hours in 6 well-plates with Lipofectamine2000 from Life Technologies and 2 µg CRISPR/Cas9 plasmids (expressing gRNA 1 and 2) or 2 µl siRNA (20 µM stock) [negative control (#4390844) and GAPDH (#4390849) from Ambion] in DMEM.

CRIPSR/Cas9 transfected cells were harvested after 72 hours and seeded in low numbers for clonal growth. Clones were picked and scaled-up to 6 well-plate size for subsequent genotyping. A genotyping PCR was performed with primers (5′-GACACTTTTGGGCTCTTTGC-3′, 5′-AACTCTCCTCGGGCACTTCT-3′) flanking the region in which both gRNAs target on genomic DNA which was extracted using the QIAquick PCR Purification Kit. A smaller amplicon due to an introduced deletion was distinguished from wild type alleles with electrophoresis using a 2% agarose gel. Clones with heterozygous deletions were retargeted by repeating the transfection and cloning procedure to increase the chance for clones with homozygous deletions. Sanger sequencing was applied to determine the exact deletions on sequence level.

For transfections with siRNAs, cells were lysed after 48 hours for Western blot analysis with RIPA buffer (Tris HCl pH 8.0 10 mM, EDTA 1 mM, EGTA 0.5 mM, SDS 0.1%, deoxycholic acid 0.1%, NaCl 140 mM, Triton X-100 1%, 1x PIC).

### Stable isotope labelling by amino acids in cell culture (SILAC)

For SILAC experiments, epidermal stem cells were cultured for 6 days in R/K-deficient SILAC FAD medium (DMEM:F12 for SILAC, Thermo Scientific #88215 mixed 1:1 with R/K-deficient DMEM, Gibco A14431-01) supplemented with 10% dialyzed serum (Sigma-Aldrich, F0392) and 800 mM L-Lysine ^13^C_6_^15^N_2_Hydrochloride and 482 mM L-Arginine ^13^C_6_^15^N_4_ hydrochloride (Sigma-Aldrich) for “heavy”-labeled media or 800 mM L-Lysine ^12^C_6_^14^N_2_-Hydrochloride and 482 mM L-Arginine ^12^C_6_^14^N_4_ hydrochloride for “light”-labeled media^27^.

### Antibodies

AGO2 was detected with anti-AGO2 clone 11A9 (#SAB4200085, Sigma-Aldrich), anti-AGO2 (#Abcam571130), anti-AGO2 (4FG, sc-53521) and as negative control antibodies were used anti-FLAG (f1804, Sigma-aldrich) and rat IgG isotype 2a (#54447, Novus biologicals). For loading controls, GAPDH and ATP synthase β were used with anti-GAPDH (Abcam8245) and anti-ATP synthase β in the same dilution as described^37^. H3K4me3 on chromatin was detected with anti-H3K4me3 (Abcam8580). SMARCC1/BAF155 detection occurred with anti-BAF155 from Bethyl laboratories (#A301-019A). Purified Rabbit IgG antibody (#P120-101, Bethyl laboratories) was used as negative control. For detection on Western blot, secondary antibodies were used from Licor (IRDye 800CW Goat-anti-Rat Antibody, IRDye 800CW Goat-anti-Rabbit Antibody and IRDye 680RD Goat-anti-Mouse Antibody) and from Dako (rabbit anti-rat immunoglobulins/HRP, #P0450).

### ChIP-qPCR and ChIP-MS

Cells (7.5 × 10^7^) were cross-linked in suspension with 1% formaldehyde in PBS for 10 minutes, quenched for 5 minutes with 1.25 M glycine in PBS and washed in PBS. Cross-linked cells were incubated 4 hours in lysis buffer (5 mM Tris-HCl pH 8.0, 85 mM NaCl, 0.5% NP40, 1X PIC) on ice, following 50 strokes with a dounce homogenizer to enrich for nuclei. Nuclei were sonicated in sonication buffer (50 mM Tris-HCl pH 8.0, 10 mM EDTA, 0.1% SDS, 150 mM NaCl and 0.5% deoxycholic acid) to get an average chromatin fragment length of 500 bp. Overnight, 15 µg antibody was incubated following a bead capture step of 4 hours with 100 µl protein G-coated magnetic beads (life Technology). Subsequently, beads were washed 5X in RIPA buffer. From this point, the sample was prepared for qPCR and next-generation-sequencing or quantitative mass spectrometry (described below). For sequencing and qPCR, chromatin was reverse cross-linked overnight at 65 °C followed by a 1 hour incubation step with proteinase K (1 µg/µl) and RNAse A (1 µg/µl) at 37 °C with. The DNA was purified with the Qiaguick PCR purification kit upon. Quantitative PCR was performed with Sybr green (Bio-Rad) and custom primers around the negative control region (5′-ATATCAAGGGCTCAGTGTTGGT-3′, 5′-GTGGAGGGATGGAGGTTATACA-3′) and the transcription start sites of HNRNPA2B1 (5′-CGCAAGGCAGGTGAGAAAC-3′, 5′-CGGAAAACAAAAAGGGAAGG-3′), DICER1 (5′-GCCTCCATTGTTGCTCCTT-3′, 5′-GCGGAAGTGGGTGTTTGTT-3’), GSK3β1 (5′-GCTGGCTGGAGGAGACATT-3′, 5′-TTCACCAATCACCGAAGGA-3′) and Meis1 (5′-GCCTCCTGAACCTTCTTTCTCT-3′, 5′-AATGGGGTAGATCGTCGTACTG-3′).

### Next-generation-sequencing and analysis

Using the KAPA Biosystems kit (#KK8504), between 0.5 and 5 ng ChIP-derived DNA was trimmed, A-tailed, provided with Nextflex adaptors and amplified with 10 PCR cycles. Subsequently, DNA was size-selected with the E-Gel® iBase™ Power System (Invitrogen) to purify for fragments between 300 and 400 bp. The libraries were quantified on the Agilent 2100 Bioanalyzer and evaluated by qPCR to confirm representation of enrichments at specific loci. The libraries were sequenced with the Nextseq. 500 to generate 25–45 million reads which were aligned to the human hg19 genome with the Burrows-Wheeler Alignment tool (BWA)^38^ and processed with SAMtools^39^ to generate BAM files. Peaks were called from BAM files using “Model-based analysis of ChIP-Seq version 1.4” (MACS14)^40^ with a P-value cut-off of 1 × 10^−8^. The called peaks were manually checked with BigWig tracks in IGV from Broad institute and visualized with UCSC genome browser. Overlaps with specific chromatin states of the normal human epidermal keratinocyte (NHEK) genome were analyzed with BEDtools^41^ using the wgEncodeBroadHmmNhekHMM.bed file from ENCODE^32^.

### Immunoprecipitation, sample preparation and quantitative mass spectrometry

Cells were washed with PBS and lysed on 15 cm plates with 1.5 ml RIPA buffer. When indicated in the results section a less stringent lysis buffer was used (Tris HCl pH 7.5 50 mM, Triton X-100 0.25%, glycerol 10%, NaCl 150 mM, 1x PIC). Lysate was centrifuged 10′ at 17,000 g to remove precipitates. Overnight, 1 ml lysate was immuno-precipitated with either 6 µg anti-AGO2 11A9, 1 µg anti-BAF155 A301-019A, 6 µ anti-IgG (rat) or 1 µg anti- IgG (rabbit). Antibody precipitates were captured with 50 µl protein G-coated magnetic beads (life Technology) for 4 hours and subsequently washed 5X in RIPA buffer or the less stringent lysis buffer when indicated.

Beads loaded with immuno-precipitates from (Ch)IP experiments were washed 2X with 100 mM NH_4_HCO_3_. Heavy and light labeled SILAC samples of different ChIP experiments were combined after the first wash step. Proteins were on bead digested with 15ul trypsin (10 µg/ml in 100 mM NH_4_HCO_3_) overnight. Supernatant was taken and the digestion was stopped by adding up to 100 µl with triethylammonium bicarbonate (100 mM) including 5% formic acid. For quantification with dimethyl-labeling, light- or intermediate dimethyl labels were added at this point^31^. Briefly, 4 µl of 4% label and 4 µl of 0.6 M NaBH_2_CN were added and incubated for 1 hour. Before merging heavy and light labelled samples, 16 µl 1% ammonia was added and after merging 10ul of 100% trifluoric acid. Before sending the samples for Mass spectrometry analysis, a purification was applied on StageTips^42^. Peptides were loaded onto a pulled fused silica column (New Objectives) packed in house with 1.8 μm Reprosil-Pur C18-AQ (Dr. Maisch, 9852). Using the Easy-nLC 1000 (Thermo Fisher Scientific), peptides were separated in a 120 minutes gradient and directly injected into a QExactive mass spectrometer (Thermo Fisher Scientific). The mass spectrometer was operated in TOP10 data dependent acquisition. Full MS were recorded at a resolution of 70,000 at m/z = 400 and a scan range of 300–1,650 m/z. MS/MS spectra were recorded at a resolution of 17,500.

### Mass spectrometry data analysis

Raw mass spectrometry data analysis was analysed with the MaxQuant software package, version 1.3.5.7, using standard settings with the additional option “match between runs”^43^. For label free quantification, LFQ and iBAQ were selected. The output proteingroupst.txt table was filtered for contaminants, reverse hits, number of unique peptides (>0) and number of peptides (>1) in Perseus (MaxQuant package) and R as described. For Label dependent quantification, the ‘ratio H(eavy)/L(ight) normalized’ was taken from two replicate experiments (reverse and forward labelled) and plotted in Excel to visualize fold enrichments. For label free interactor identification, IPs were applied in triplicate to allow t-test based statistics on LFQ values and stoichiometry analysis on iBAQ as described earlier^33^. In Perseus, the option imputation of missing values was applied by normal distribution with default settings. Statistical outliers for the pull down of AGO2 11A9 or BAF155 A301-019A were determined with a two-tailed t-test and multiple testing correction was done with a permutation based false discovery rate method in Perseus. Stoichiometry analysis of AGO2 and SMARCC1 was determined by comparing the relative abundance of the identified interactors as measured by the iBAQ values. The iBAQ values of the proteins in the IgG IP (control) samples were used as background binding level and were subtracted from iBAQ values from the bait IP samples. The relative abundance value of each interactor was scaled to the bait protein which was set at 1.

### Western blot

Beads loaded with immuno-precipitates from IP experiments were incubated 10 minutes at 95 °C in 100 µl 1X Sample buffer (1% SDS, 40 mM Tris HCl pH 6.8, 5% glycerol, 0,25% β-ME, BPB) to release and denature the proteins. Whole cell lysates were incubated with 4X Sample buffer. Proteins were separated on standard 4–15% gradient gels (Bio-Rad) and blotted on a PVDF membrane using the Bio-Rad Trans-Blot^®^ Turbo^TM^ RTA Transfer Kit. Blots were blocked 30 minutes. with 3.5% milk in PBS (w/v) and stained with primary antibody in blocking buffer overnight at 4 °C (AGO2 11A9 1:500, BAF155 A301-019A 1:500, GAPDH Abcam8245 1:2500 and ATP synthase β 1: 10,000). After primary antibody incubation, blots were washed 3X with TBST, stained with secondary antibodies for 1 hour at room temperature and washed again 3X with TBST. Detection occurred using the Odyssey CLx (near-infrared fluorescence) or the LAS4000 (chemiluminescence).

### Cloning CRISPR/Cas9 constructs

PX330 based constructs were generated as described^44^. Briefly, guide RNAs (gRNAs) were designed to target the anti-AGO2 11A9 epitope-encoding region of AGO2 with the online Zhang lab CRISPR design tool (http://tools.genome-engineering.org). Oligo’s encoding the gRNAs were obtained from Biolegio (Nijmegen, The Netherlands), representing the forward and reverse sequences 5′-CACCGGCGCCGCCGCCCCCCATCCA-3′ and 5′-AAACTGGATGGGGGGCGGCGGCGCC-3′ for gRNA1 and 5′-CACCGAGAGAGAACACCCATTAACG-3′ and 5′-AAACCGTTAATGGGTGTTCTCTCTC-3′ for gRNA2. Top and bottom oligo’s were annealed and ligated into BbsI digested pX330-U6-Chimeric_BB-CBh-hSpCas9 (Addgene #42230) upon poly nucleotide kinase and Antarctic phosphatase treatment of the annealed oligo’s and backbone respectively. Identification of the correct constructs was achieved with a colony PCR in which the U6 primer (5′-GAGGGCCTATTTCCCATG-3′) acted as forward primer and the reverse gRNA oligo as reverse primer. Constructs were subsequently amplified in E. coli strain DH5α.

### Data availability

The datasets generated during and/or analysed during the current study are available from the corresponding author on reasonable request.

## Electronic supplementary material

Supplementary Information

Supplementary Dataset 1
