# Supplementary material for: Reactivity of human AGO2 monoclonal antibody 11A9 with the SWI/SNF complex: A case study for rigorously defining antibody selectivity
Source: Sci Rep. 2017 Aug 4;7:7278. doi: 10.1038/s41598-017-07539-4 (PMC5544689; doi:10.1038/s41598-017-07539-4)
Supplement: Supplementary file 1 — Supplementary Information [file 41598_2017_7539_MOESM1_ESM.pdf]

# Reactivity of human AGO2 monoclonal antibody 11A9 with the SWI/SNF complex: A case study for rigorously defining antibody selectivity.

Roderick A.P.M. van Eijl[1], Teun van den Brand[1], Luan N. Nguyen[2] and Klaas W. Mulder[1,*]

Radboud University Nijmegen, Department of Molecular Developmental Biology[1] and Molecular Biology[2], Radboud Institute for Molecular Life Sciences, The Netherlands

* To whom correspondence should be addressed. k.mulder@science.ru.nl

running title:
**AGO2 antibody associates with the SWI/SNF complex.**

Keywords:
11A9 AGO2 antibody, AGO2, SMARCC1, AGO2 Knock-out, Mass spectrometry, ChIP sequencing

## Supplementary Information

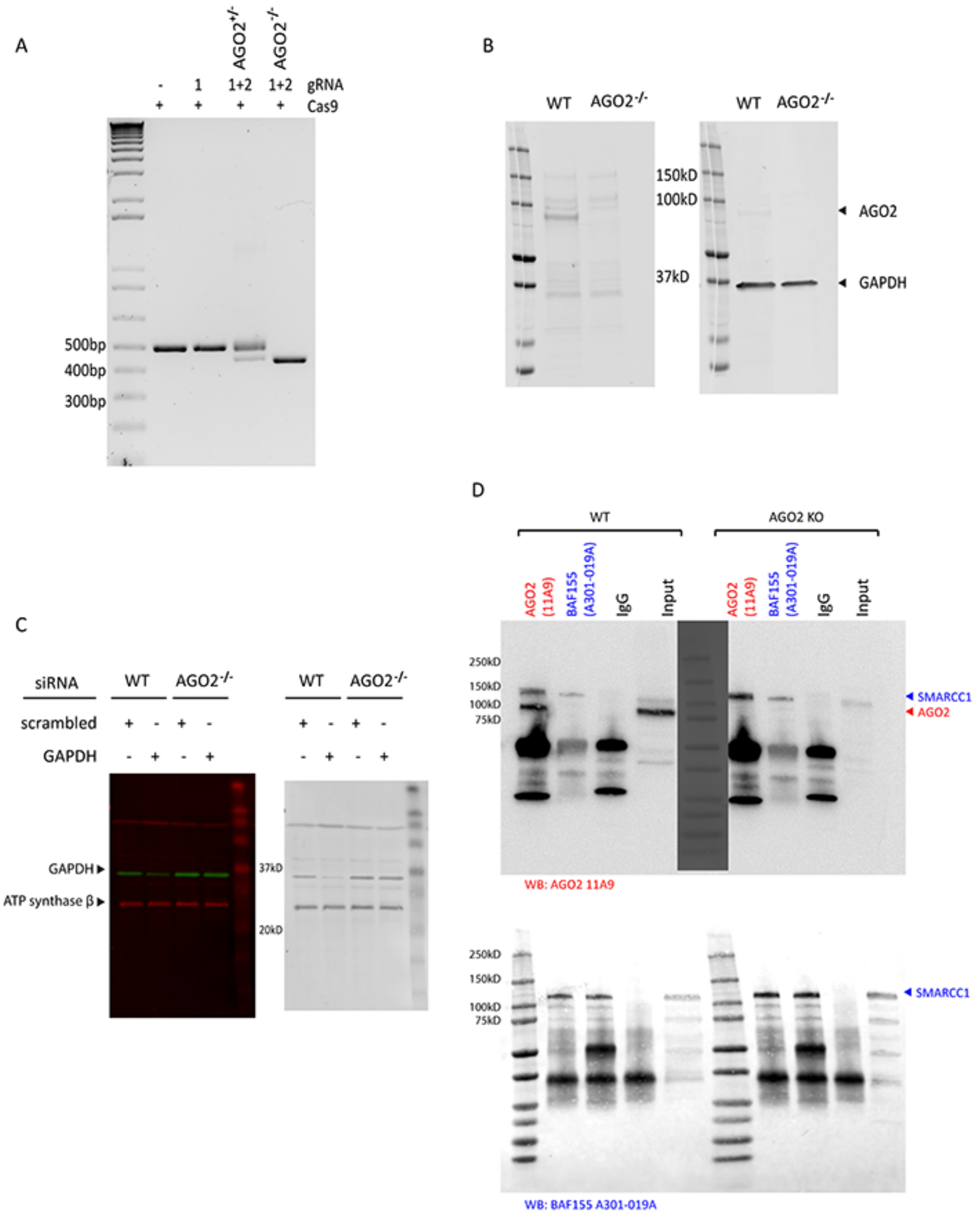


**Supplementary Figures.** Non-cropped images of gels and Western blots. (A) Uncropped agarose gel image of figure 4A showing the CRISPR-Cas9 targeted cells. (B) Uncropped Western blot of figure 4C stained with anti-AGO2 11A9 (rat) and anti-GAPDH (mouse) loaded with WT and AGO2 knock-out HEK293T cells. (C) Uncropped Western blot from figure 4D in colour (distinguishing GAPDH and ATP synthase β) and grey as in figure 4D. The western blot was loaded with lysates derived from WT and AGO2 knock-out HEK293T cells treated with siRNAs. (D) Western blot from figure 6F loaded with Immuno-precipitates

stained with anti-AGO2 11A9 and anti-BAF155. To allow detection of both proteins separately on the same Western blot, detection of anti-AGO2 was performed on the LAS4000 (chemiluminescence) and detection of BAF155 on the Odyssey CLx (near-infrared fluorescence).
